# Positive effect of a diggable substrate on the behaviour of a captive naked mole rat colony

**DOI:** 10.1038/s41598-024-64146-w

**Published:** 2024-08-29

**Authors:** Myriam Amari, Alma Mary, Pauline Zablocki-Thomas, Aude Bourgeois, Emmanuelle Pouydebat

**Affiliations:** 1 UMR 7179 MECADEV, CNRS/MNHN, Département Adaptations du Vivant, Mécanismes Adaptatifs et Evolution, Muséum National d’Histoire Naturelle, 57 Rue Cuvier, 75231 Paris, France; 2 Département de Biologie, École normale supérieure, PSL Université Paris, 75005 Paris, France; 3 Westfälische Wilhelms-Universität Münster, Münster, Germany; 4 Ménagerie, Le Zoo du Jardin des Plantes, Muséum National d’Histoire Naturelle, 57 Rue Cuvier, 75005 Paris, France

**Keywords:** Zoology, Animal behaviour, Ecology, Behavioural ecology

## Abstract

Naked mole rats (*Heterocephalus galber*) are eusocial mammals from East Africa. Their extraordinary social organisation is accompanied by remarkable adaptations to an underground lifestyle, extreme longevity and resistance to many diseases, making naked mole rats a highly relevant model for biological research. However, their living conditions in controlled environments do not allow them to express fundamental behaviours: digging galleries and exploring. This gap probably constitutes a bias to any behavioural or even medical study, because it represents a potential obstacle to their well-being. In this article, we tested the effects of the introduction of a diggable substrate on the behaviour of a colony of naked mole rats at the Menagerie, le Zoo du Jardin des Plantes, Paris. We measured individual exploratory latencies, the number of entries per minute and the frequency with which naked mole rats gnawed tunnels during observation trials. We found that: (i) young individuals explore more quickly, (ii) the introduction of a diggable substrate encourages exploration and digging behaviour, and (iii) could therefore be a relevant element to introduce under human care. This new environmental design could improve the welfare of naked mole rats by creating opportunities for cognitive challenges such as exploration and environmental control.

## Introduction

Exploratory behaviours allow animals to gather information about their environment and reduce uncertainty within it^1^. Exploration can be influenced by the physical environment, but also by how it is perceived by the animal. For example, social organisation, motivation, cognition, memory^2^ and internal states such as anxiety can influence exploration. Indeed, less stressed animals have a lower exploratory latency (the time required for an animal to begin exploring a new area or object) than those more stressed ones in a wide variety of situations^3^. Thus, a reduction in exploratory latency after the introduction of a change in the environment could indicate a reduction in stress^3^. By measuring exploratory behaviour, individual personalities can be characterised, emotions assessed and the links between personality and morphology studied. Here we have focused on an animal model that is relevant to many medical studies, but for which the application of behavioural knowledge to management is still inadequate: naked mole rats.

Naked mole rats (*Heterocephalus galber*) are eusocial mammals from East Africa. They exhibit eusocial behaviour by living in castes consisting of fertile and non-fertile individuals, including a reproductive queen and generally one reproductive male, although sometimes multiple males can reproduce^4^. Unlike many eusocial Hymenoptera, they do not exhibit morphologically distinct casts among the non-breeding workers^5,6^. All specialisations are behavioural and vary along a continuum, from defence to occupational activities and offspring care^7–10^. Recent in-depth studies have revealed that tasks such as pup care, colony defence and dispersal behaviour are unequally distributed^10,11^. Age emerges as a key factor in the distribution of tasks, with older individuals being involved in colony defence and younger ones in pup transport^10^ and burrowing^12^. Overall, non-breeding individuals vary in their cooperative investment but do not specialise in specific tasks. Their behavioural organisation is closer to other cooperatively breeding vertebrates such as meerkats than to eusocial insect species^12^.

Although their extraordinary social organisation is accompanied by remarkable adaptations to the subterranean lifestyle^13–15^, their living conditions in captivity often do not allow them to express a fundamental behaviour: digging galleries and exploring, which could jeopardise their welfare. Even though digging substrate as enrichment for captive mole-rats has a long history, it is still not generalized enough. Back in the 1980s, Brett et al. introduced a burrow that returned the sand dug out of the tunnels to a different tunnel, and Braude et al. used cork for digging substrate because it did not create mud that would scratch or stick to the clear plastic tubing^16^. Other researchers used peat as a substrate^17^. Nevertheless, besides sticks, packages, or thin pieces of clay used as enrichment in zoos, the use of a permanent digging substrate providing frequent digging opportunities and allowing naked mole rats to explore and dig shape their environment is not mandatory, nor common. The impossibility of digging could be a source of frustration and possibly stress. Stress, including that caused by new odours or handling, can in turn be a source of conflict in the colony, leading to putsches in which the queen is killed by another female (observed by the veterinary team) or to a peak in mortality^18^. It is therefore essential to find ways of reducing stress and frustration in order to promote the well-being of naked mole rats in captivity.

As stated by Poole in 1997^19^, « happy animals make good science ». Indeed, if standardisation and impoverishment of laboratory animals’ living conditions was an initial measure to lower data variability, several studies have showed that enriched or heterogenized environments decrease variability and improve data reproducibility^20^, as well as the poor translational rate of preclinical research^21,22^. However, many laboratory animals used in fundamental and medical research like rats and zebrafish are still denied fundamental behaviours like hiding or burrowing no matter how crucial these behaviours are^23^.

The concept of animal welfare encompasses both the physiological and psychological health of animals, as set out in the Five Domains model^24^. In this framework, good welfare conditions can be achieved when an animal is free to adapt to external or internal factors and constraints by adopting species-specific behaviours and achieving a positive mental state^24^. For example, giving captive animals control over their environment, a property known as 'agency', and the choice to adopt species-specific behaviours can trigger positive mental states and promote well-being. However, performing a species-specific behaviour does not mean reproducing all the natural behaviours exhibited by a species in the wild. Indeed, natural behaviours are not always relevant in captivity (e.g. being chased by a predator), and they cannot be used as a measure of well-being since they fail to take into account what animals want, which is ultimately what motivates their behaviour^25^. As a result, animals may adopt unnatural behaviours to obtain a similar reward. For example, when given the choice between an automatic brush and trees to rub against, cows systematically chose the automatic brush and were willing to open much heavier doors to reach the automatic brushes than to reach the trees^26^. What matters is not the rubbing behaviour, but the result of the rubbing, i.e. the associated reward of touch.

Naked mole rats are rodents that have gained importance as a biomedical research model for various conditions including hypoxic brain injury, cancer and nociception^27^, and much is unknown about how to optimise housing conditions in captivity and rather little, is done to provide them with the opportunity to engage in species-specific behaviours, like digging, which could be detrimental to their welfare. We decided to investigate the issue of lacking digging opportunities by studying a captive colony of naked mole rats at La Ménagerie, le Zoo du Jardin des Plantes, Paris. This colony had never interacted with a diggable substrate before.

We asked a simple question: does the introduction of a diggable substrate modify the behaviour and organisation of the colony, and is it beneficial to their well-being? To answer this question, we compared exploratory behaviours and gnawing frequencies of the colony before and after the introduction of substrates designed to promote digging in reptiles. During our experiments, we guaranteed individuals a permanent contact with their colony, and allowed them to choose to participate in or retreat from the experiment by only adding new tunnels parallel to their usual circuit. Their usual circuit consisted of two central tunnels of different diameters (5 cm and 8 cm). Before introducing new substrates, in what we called “Phase A”, we added downward or upward one-way tunnels (respectively conditions “no substrate, upward angle” and “no substrate, upward angle”) and measured individual exploratory latencies. Then, we introduced new substrates (“New substrates” phase) by filling downward tunnels to observe first interactions with the reptile substrates, and check if everything went well. The colony then had several days of free access to the substrates, and we proceeded to “Phase B” where we measured exploratory latencies again, either with an empty upward tunnel (condition “post substrate, upward angle”) or a half full tunnel to investigate the immediate effect of the substrate (“substrate, upward angle”). Additionally, naked mole rats were observed to repeatedly destroy their boxes by gnawing on the tunnel walls and box angles, which sometime jeopardise their safety. This behaviour is essential for wearing down incisors^28^, but might be a redirected behaviour and reflect a lack of digging opportunities. In order to assess whether gnawing on the walls was affected by a longer-term opportunity to dig into a substrate, we compared observational sessions of behaviours in the two main tunnels of the colony, before (“Phase A”) and after (“Phase B”) exposition to the substrate. In the latter case, a third wide and horizontal tunnel full of substrate was added during the observational session. Our hypothesis was that the introduction of the substrate would decrease exploratory latencies and increase activity of individuals in the colony. We also expected gnawing on the walls behaviour to decrease.

## Results

### Direct observations: interactions with the substrate

**Figure 1:**
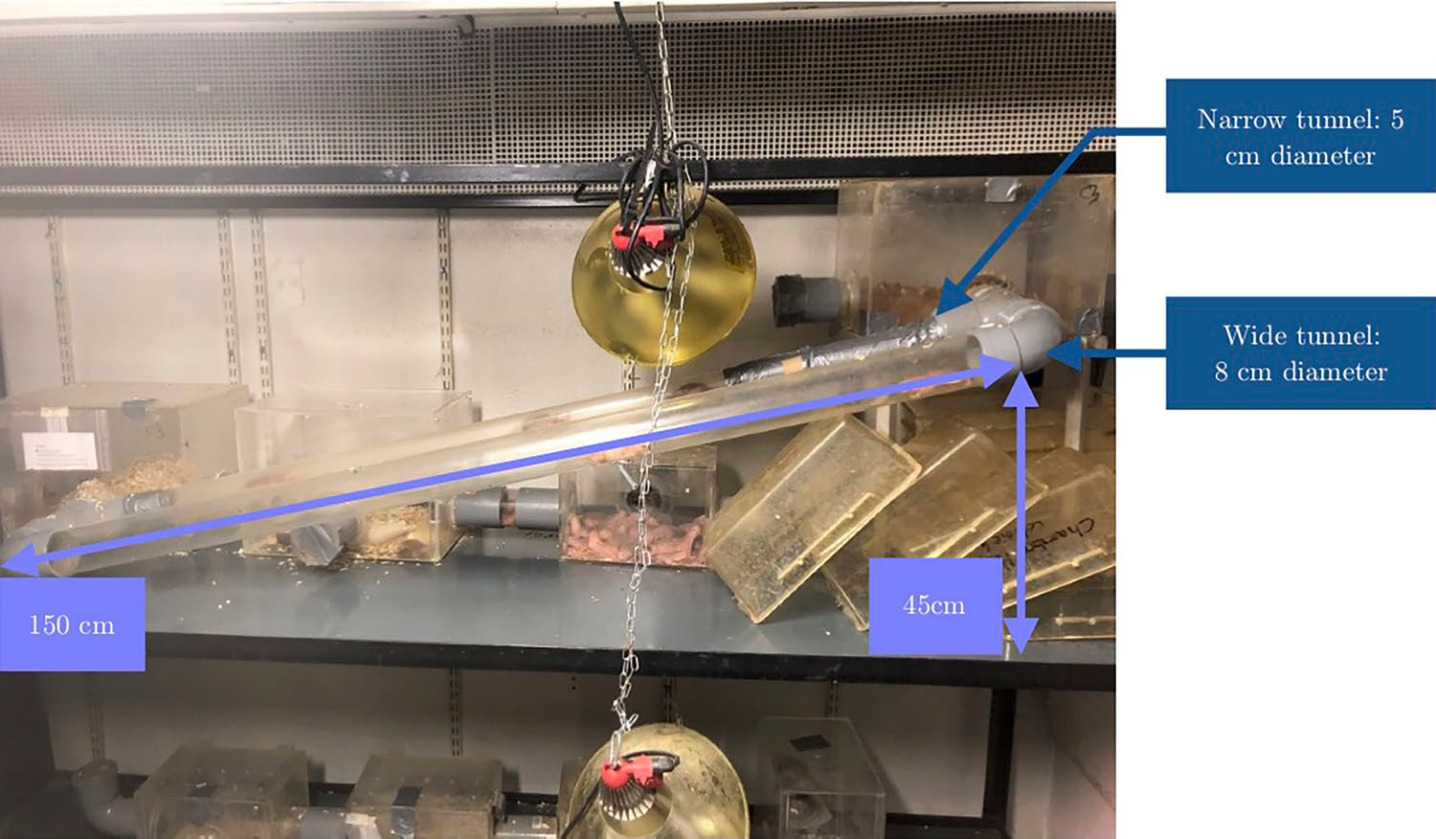
Extracts from the films of the introduction of the new substrates (**a** and **c**) and the time budget sessions (**b** and **d**). Naked mole rats were observed to dig alone (**a**) or several at a time (**b**, **d**) and to nibble or sweep the debris (**c**).

When exposed to tunnels filled with a diggable substrate, naked mole rats quickly started to gnaw, and did so continuously until they finished the tube, or until the end of the session. They dug alone or several at a time (Fig. 1). In the adjacent tunnels, other colony members were sweeping the wastes or transporting the debris to the toilet chamber. Occasionally, they nibbled the desiccated clay.

### Characterising the most exploratory individuals: age and mass effect on presence in the tunnels

The first analysis compared the presence of individuals in each test phase with a GLMM model (Table 1). We found that the number of sessions in which an individual was present was affected by the variables mass (*ß* = 0.057 ± 0.023, *P* = 0.0121), age (*ß* = − 0.024 ± 0.0054, *P* < 0.01), and the conditions “no substrate, upward angle” (*ß* = − 2.00 ± 0.31, *P* < 0.01), “substrate, upward angle” (*ß* = 0.54 ± 0.24, *P* = 0.027) and “no substrate, downward angle” (Intercept = − 1.81 ± 0.75, *P* = 0.015). Our model was significantly better than the null model (Chi-square test: *X*^2^_6_ = 100.6, *P* < 2.2e−16) and obtained a marginal R^2^ of 0.252.

**Table 1:** Results of the global linear model with mixed effect of presence during tests.

Response variable	AIC	Predictor variable	Estimate ± SE	df	*P*
Presence (0 or 1)	885.4	–			
Presence (0 or 1)	796.8	Intercept (“no substrate, downward angle” for females)	− 1.81 ± 0.75		**0.0154**
		Mass	0.057 ± 0.023		**0.0121**
		Sex (male)	0.40 ± 0.35		0.2428
		age	− 0.024 ± 0.0054		**< 0.001**
		Condition “no substrate, upward angle”	− 2.00 ± 0.31		**< 0.001**
		Condition “post substrate, upward angle”	0.23 ± 0.25		0.3507
		Condition “substrate, upward angle”	0.54 ± 0.24		**0.0265**

Bold results are significant results with alpha = 0.05.

### Number of explorers

**Figure 2:**
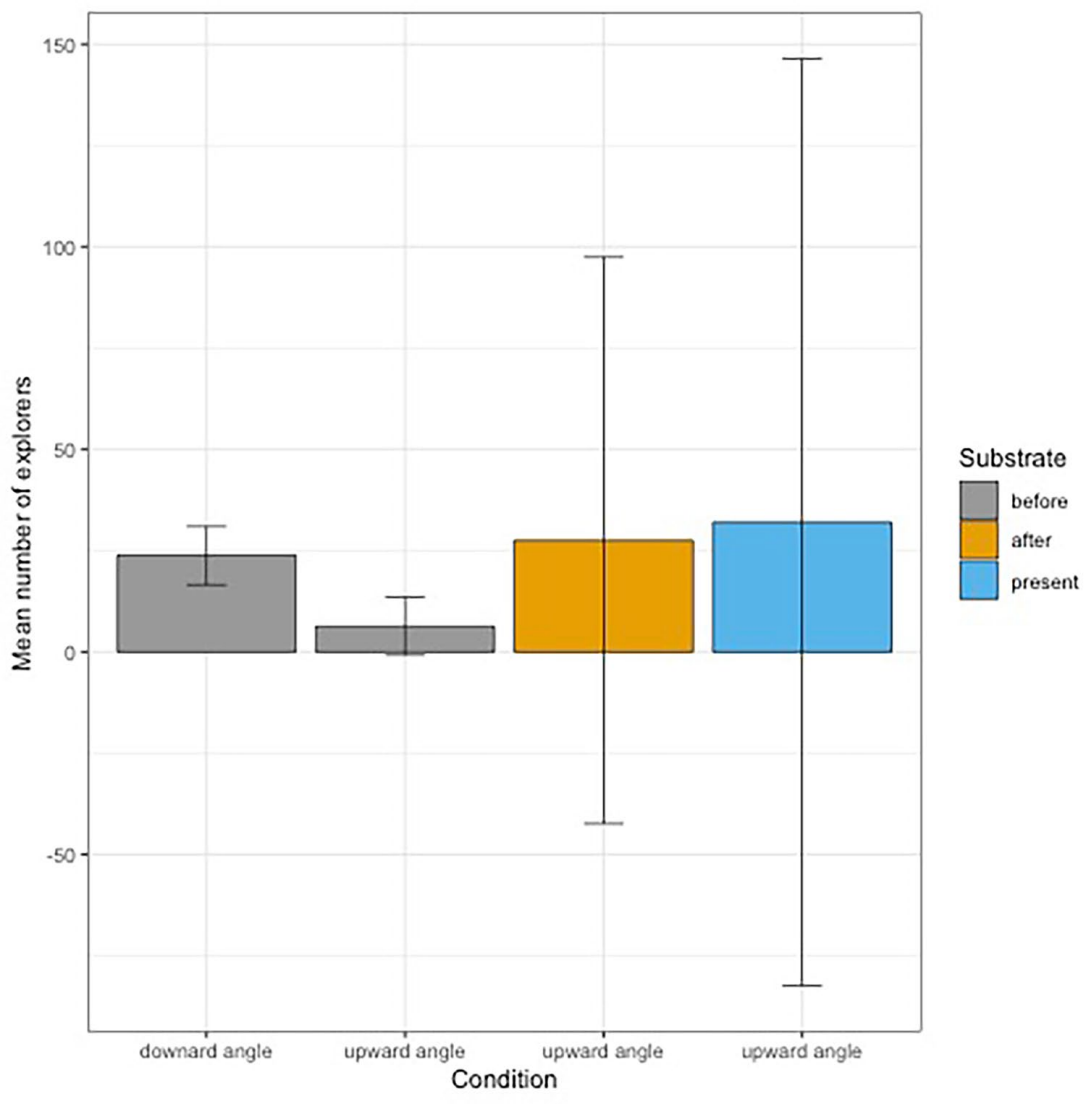
Barplot of the mean number of explorers per condition. Error bars represent 95% confidence intervals.

Based on the total number of explorers present during each session, we conducted a GLM analysis (Table 2) to test the effect of tunnel condition on the number of explorers. We found a significant effect of the condition “no substrate, downward angle” (Intercept = 3.16 ± 0.10, *P* < 0.001), and a negative effect of the condition “no substrate, upward angle” (*ß* = − 1.32 ± 0.25, *P* < 0.001). The other conditions did not significantly differ from the condition “no substrate, downward angle”. Our model was significantly better than the null model (Chi-square test: *X*^2^_3_ = 57.392, *P* = 2.119e−12) and obtained a marginal R^2^ of 0.996. The mean entries and size effects are reported in Table 3 and presented in Fig. 2.

**Table 2:** Results of the global linear model number of explorers per test.

Response variable	AIC	Predictor variable	Estimate ± SE	df	*P*
Number of explorers	123.95	–			
Number of explorers	84.082	Intercept (“no substrate, downward angle” for females)	3.16 ± 0.10		**< 0.001**
		Condition “no substrate, upward angle”	− 1.32 ± 0.25		**< 0.001**
		Condition “post substrate, upward angle”	0.15 ± 0.17		0.3869
		Condition “substrate, upward angle”	0.30 ± 0.16		0.0652

Bold results are significant results with alpha = 0.05.

**Table 3:** Mean number of explorers during the different test conditions.

Condition	Substrate	Mean number of explorers±SE	Size effect compared to condition “no substrate, downward angle”
“no substrate, downward angle”	before	23.75 ± 2.25	0.00
“no substrate, upward angle”	before	6.33 ± 1.67	4.42
“post substrate, upward angle”	after	27.50 ± 5.5	0.68
“substrate, upward angle”	present	32.00 ± 9.00	1.10

### Entry flux (number of entries per minute)

**Figure 3:**
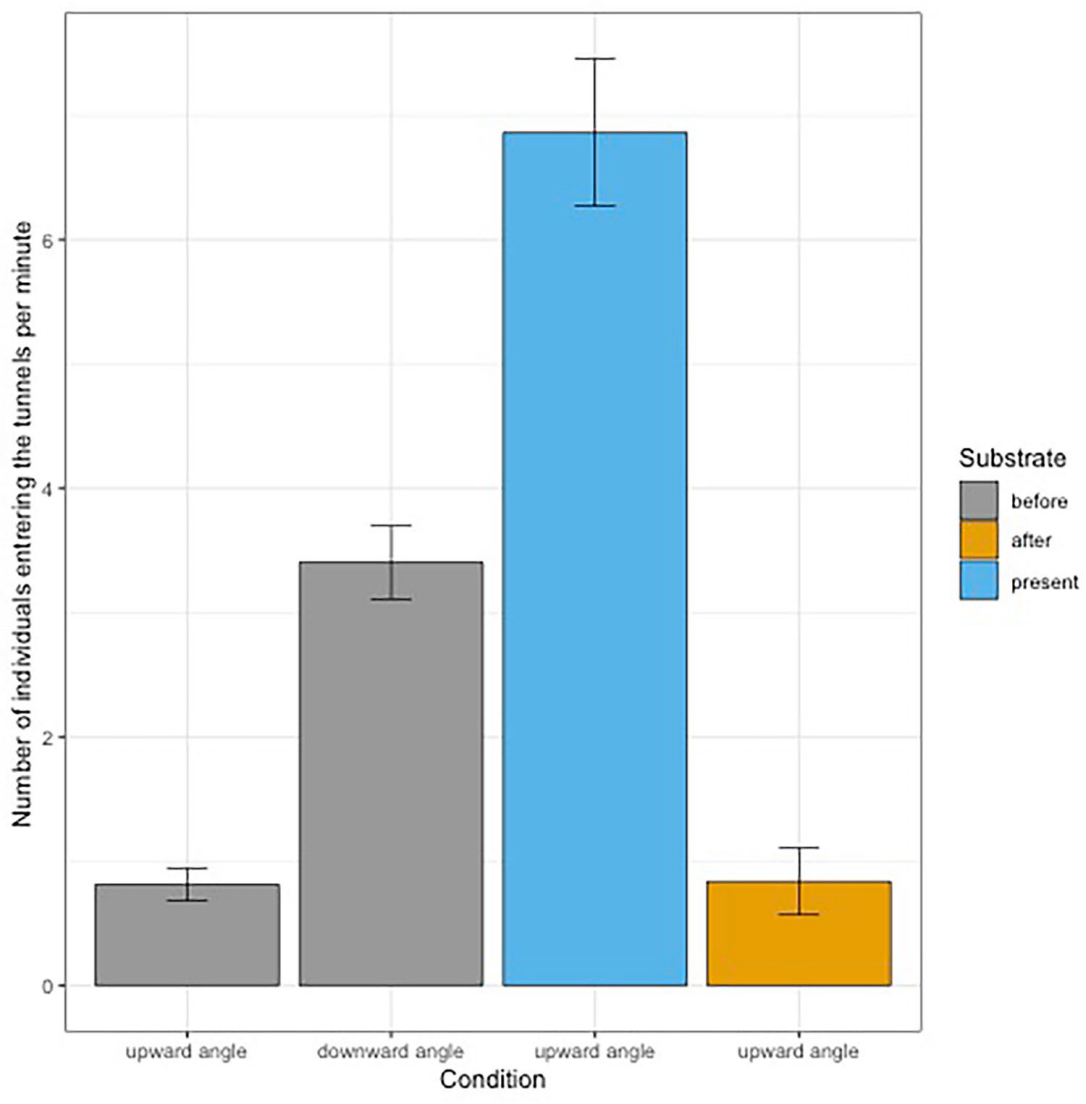
Barplot of the mean number of individuals entering the tunnel per minute and condition. Error bars represent 95% confidence intervals.

We collected data from our one-hour-long exploratory sessions and counted the number of entries by time intervals of 60 s. This led to a number of 1967 observed entries from at least 68 different individuals (4 sessions of “no substrate, downward angle”: 830 entries; 4 sessions “no substrate, upward angle”: 198 entries; 2 sessions “post substrate, upward angle”: 102 entries; 2 sessions “substrate, upward angle”: 837 entries). To test the effect of the substrate introduction on the entry flux of naked mole rats in the exploration tunnel, we conducted a GLMM analysis with a Poisson distribution (Table 4) with the tunnel condition as a fixed effect and including day, sessions, time interval and observation number (to correct for over-dispersion) as random effects. We found a significant effect of the condition “no substrate, upward angle” (Intercept = − 0.33 ± 0.13, *P* < 0.001), and positive effects of “no substrate, downward angle” (*ß* = 1.46 ± 0.18, *P* < 0.001) and “substrate, upward angle” (*ß* = 2.17 ± 0.22, *P* < 0.001). Our model was better than the null model (Chi-square test: *X*^2^_5_ = 30.448, *P* < 0.001) and had a marginal R^2^ of 0.530. The mean number of entries per minute are presented in Table 5 and Fig. 3.

**Table 4:** Results of the global linear model with mixed effect of entry flux tests.

Response variable	AIC	Predictor variable	Estimate ± SE	df	*P*
Number of entries per minute	2701.7	–			
Number of entries	2677.3	Intercept (“no substrate, upward angle	− 0.33 ± 0.13		**0.0163**
		Condition “no substrate, downward angle”	1.46 ± 0.18		**< 0.001**
		Condition “post substrate, upward angle”	0.06 ± 0.24		0.8087
		Condition “substrate, upward angle”	2.17 ± 0.22		**< 0.001**

Bold results are significant results with alpha = 0.05.

**Table 5:** Mean number of entries per minute depending on the different test conditions.

Condition	Substrate	Mean number of entries per minute ± SE	Size effect compared to condition “no substrate, downward angle”
“no substrate, downward angle”	before	3.40 ± 0.15	0.00
“no substrate, upward angle”	before	0.81 ± 0.067	1.44
“post substrate, upward angle”	after	6.86 ± 0.30	1.22
“substrate, upward angle”	present	0.84 ± 0.14	1.29

### Exploratory latencies

We collected the time of first entry for each session of each individual that participated in the test, leading to a total of 252 entries (4 sessions “no substrate, downward angle”: 85 entries; 3 sessions “no substrate, upward angle”: 38 entries; 2 sessions “post substrate, upward angle”: 55 entries; 2 sessions “substrate, upward angle”: 64 entries). By performing a GLMM with a Gaussian family, with mass, age, tunnel as fixed effects and individual identity as a random effect, we found that younger individuals had lower exploration latencies than older individuals (*ß* = 0.0088 ± 0.033, *P* = 0.008) and lighter individuals had higher latencies than heavier ones with other parameters fixed (*ß* = − 0.03 ± 0.16, *P* = 0.04). Exploratory latencies were longer in condition “no substrate, downward angle” than in condition “no substrate, upward angle” (*ß* = 0.56 ± 0.21, *P* = 0.009) and condition “substrate, upward angle” (*ß* = − 0.58 ± 0.23, *P* = 0.011) (see Table 6). Our model was better than the null model (Chi-square test: *X*^2^_5_ = 25.465, *P* < 0.001) and performed with a marginal R^2^ of 0.164.

**Table 6:** Results of the global linear model with mixed effect of the exploratory latencies during the test conditions. Significant values are in bold.

Response variable	AIC	Predictor variable	Estimate ± SE	*P*
log(latency + 0.5)	838.5	—		
log(latency + 0.5)	823.1	Intercept (Condition “no substrate, upward angle”)	7.49 ± 0.54	**< 0.01**
		Mass	− 0.033 ± 0.016	**0.041**
		Age	0.0088 ± 0.033	**< 0.01**
		Condition “no substrate, downward angle”	0.56 ± 0.21	**< 0.01**
		Condition “post substrate, upward angle”	− 0.34 ± 0.23	0.15
		Condition “substrate, upward angle”	− 0.58 ± 0.23	**0.011**

Bold results are significant results with alpha = 0.05.

### Gnawing frequency

**Figure 4:**
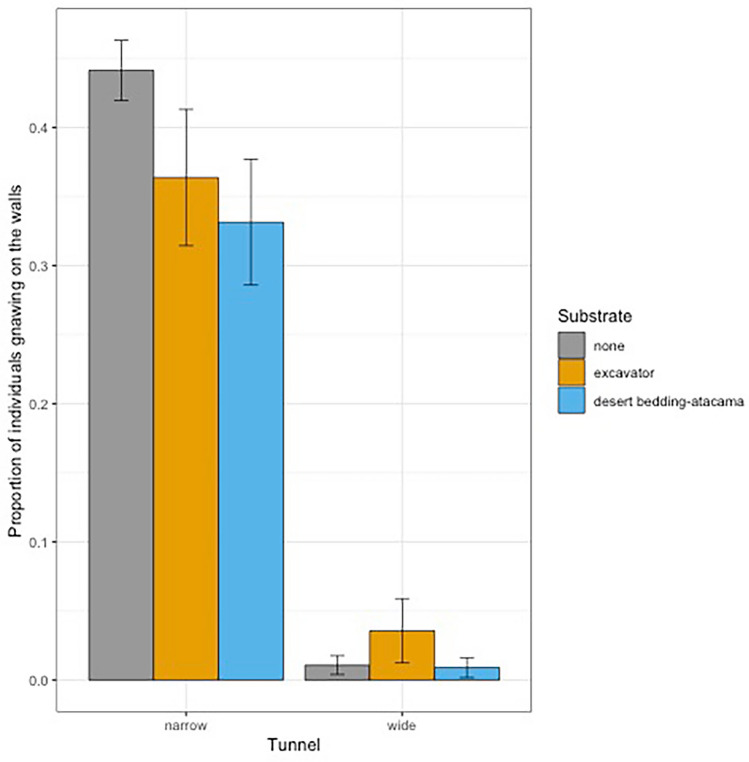
Barplot of the mean proportion of individuals gnawing on the tunnel walls per minute and tunnel. Error bars represent 95% confidence intervals.

During the two-hour-long observational sessions, we counted the number of individuals gnawing on the walls every minute, leading to a total of 1936 observations, 242 by session. We fitted global linear model with mixed effects with substrate condition (before or after substrate introduction) and tunnel condition as fixed effects, day and observation number (to correct for over-dispersion) as random effects, and a binomial family. Our model was better than the null model (Chi-square: *X*^2^_3_ = 1310, *P* < 0.01) and performed with a marginal R^2^ of 0.570. The number of individuals gnawing on the tunnel walls was higher in the narrow tunnel than in the wider tunnel (*ß* = − 4.32 ± 0.18, *P* < 0.001) and lower in presence of substrate (tendency for Excavator: *ß* = − 0.29 ± 0.27, *P* < 0.28; Excavator-Atacama: *ß* = − 0.55 ± 0.27, *P* = 0.0392) (Table 7). The mean proportions of individuals gnawing on the tunnel walls depending on the substrate exposition and the tunnel considered are presented in Table 8 and Fig. 4.

**Table 7:** Results of the global linear model with mixed effect of the proportion of individuals gnawing on the walls of the tunnels. Significant values are in bold.

Response variable	AIC	Predictor variable	Estimate ± SE	*P*
Proportion of individuals gnawing	4225.9	–		
Proportion of individuals gnawing on the walls	2921.9	Intercept (no substrate in the narrow tunnel)	− 0.25 ± 0.15	0.0925
		Wide tunnel	− 4.32 ± 0.18	**< 0.001**
		Excavator	− 0.29 ± 0.27	0.28
		Excavator-Atacama	− 0.55 ± 0.27	**0.0392**

Bold results are significant results with alpha = 0.05.

**Table 8:** Mean proportions of individuals gnawing on the tunnel walls depending on the substrate exposition and the tunnel considered.

Tunnel	Substrate	Mean proportion of individuals gnawing on the walls ± SE	Size effect compared to each tunnel without substrate	Mean number of individuals gnawing on the walls ± SE	Size effect compared to each tunnel without substrate
Narrow	None	0.44 ± 0.1	0	2.58 ± 0.07	0.00
	Excavator	0.36 ± 0.03	0.27	1.01 ± 0.07	1.05
	Desert Bedding-Atacama	0.33 ± 0.02	0.38	0.80 ± 0.06	1.27
Wide	None	0.011 ± 0.004	0	0.033 ± 0.008	0.0
	Excavator	0.04 ± 0.01	0.26	0.05 ± 0.02	0.26
	Desert Bedding-Atacama	0.06 ± 0.004	0.03	0.02 ± 0.01	0.03
Narrow vs wide			2.3		2.17

## Discussion

By measuring the number of entries per minute (entry flux) during exploratory tests, we found that entry fluxes are significantly lower in the absence of substrates before their introduction to the colony (condition “no substrate, downward angle” which also had the lowest number of different explorers, condition “no substrate, upward angle”) than in presence of substrate (condition “substrate, upward angle”) or after exposition to the substrate even without substrate (condition “post substrate, upward angle”). We did not find any significant difference between conditions “no substrate, upward angle” and “post substrate, upward angle”. Because both tunnels had the same spatial orientation, we could conclude that presenting the substrate during the preceding weeks, on its own, did not encourage individuals to explore. However, when the substrate was present, the number of individuals to enter the tunnels per minute was higher than in all other conditions but condition “no substrate, downward angle”. We can deduce that naked mole rats choose to explore the new substrate and are more active when it is present.

The downwards tunnel configuration (condition “no substrate, downward angle”) had the highest entry flux. This could be explained by a spatial orientation that is closer to their natural behaviour (digging towards the bottom), or by a least costly effort because of a downhill incline. Testing different inclinations could answer this question.

We also found that exploratory latencies are significantly shorter when the tunnel is half-full (condition “substrate, upward angle”) than when it is empty (condition “post substrate, upward angle”). Moreover, younger individuals are the first to explore, in accordance with a previous study^12^ in which the authors found that young individuals are the most active.

Our captive colony of naked mole rats had access to two central parallel tunnels differing in diameter. The narrow tunnel was about the width of two individuals, and we observed them to jostle, pass over one another and pull each other’s’ tail. In the wider tunnel, the animals occasionally huddle together and slept in a pile. This size difference thus might have behavioural and biological implications that should be tested in the future. We expected naked mole rats to be more drawn to gnawing on the walls. We did find a higher frequency of gnawing on the walls in the narrow tunnel than in the wider one, and we did find a significant decrease in the gnawing on the wall frequency in presence of the substrate. Gnawing the substrate could thus compensate for gnawing on the wall behaviour. It would be interesting to keep track of the long-term rate of damages on the walls of the tunnels and chambers to see whether the presence of the substrate did decrease the rate of gnawing behaviour. Indeed, three months after the end of the study, the keepers had already observed a decrease in such damages.

Our study enabled us to show a decrease in a naked mole rat colony means exploratory latency in presence of a diggable substrate. We suggest the colony showed an increased motivation to be active since: (i) it organised around the task with individuals digging and others sweeping the debris, (ii) it increased the behavioural diversity of the colony—which has been pointed out as a potential indicator of welfare^29^—by providing opportunities to dig, (iii) many individuals chose to dig on their own will, and providing the opportunity to choose has been shown to be valued by animals^30^.

However, our study presents limitations. First, we conducted our experiment on only one captive colony, and we would need to replicate such experiments on other colonies. This raises the question of generality of our results, and limits the inference of our statistics. Second, we were not able to identify individuals at all times, which reduced the potential scope of our study. For example, since we could not observe the whole colony, nor a small sample of it to conduct a real time budget, we had to focus on the central tunnels only. When we introduced the third tunnel so that the animals would dig in it, we could not see how many of them, and which of them, were actually digging. We only had access to the number of individuals in the other two tunnels, and how many of them were gnawing on the wall. Since there were fewer individuals in those tunnels when the substrate was present, even a few digging events made the frequency rise. To confirm by observation the effects of individual characteristics, and deepen our understanding of the colony organisation, a better tracking system could be used. For example, smaller microchip scanners could be stuck inside the tunnels and connected to a computer to automatically record entries and exits of individuals. This could even allow us to gather information on duration of exploration.

It would be relevant to collect reproductive success data and behavioural data such as conflicts on the long term, since previous litters did not survive (probably killed by the colony members) and recurrent injuries happen. Our results could also be discussed with other researchers who have been managing captive colony with other kinds of digging enrichment, on the long term.

When assessing the value of an enrichment, it is important to see if animals choose to use an enrichment and measure how much they value it in terms of effort they are willing to invest to gain access to it. Here, the colony had the choice to explore tunnels, whether empty or full of substrate, and they chose to do so continuously until they had dug through the tunnel. They did so even though digging and sweeping debris is costly in itself. To prove that naked mole rats value the digging opportunity, we should aim to measure the effort they are willing to invest to dig. For example, we could use substrates with drastically different hardness, or play on the tunnel’s incline. Naked mole rats could be intrinsically motivated by digging since as they go, they modify their environment and receive feedback that could be perceived as a positive reward. It would also be of great interest to investigate what substrate naked mole rats would choose to dig in if they had the choice. Cork, as used by Braude^28^, might also be a good compromise to make it easier for the care-staff to clean the tunnels and avoid skin desiccation. Permanently exposing naked mole rats to a diggable substrate could thus be a realistic solution to favour their exploratory and digging behaviours in captivity, and improve their welfare conditions. Such insight could also be beneficial for medical and fundamental studies by improving reproducibility and translation rate to humans through better welfare of naked mole rat colonies raised in laboratories.

Additionally, a similar experiment had been conducted by Sherman and Lacey^16^. They introduced three captive naked mole rat colonies with fresh dirt packed into tunnels so they could observe and score digging behaviour. Their focus was on workload distribution and they measured which individuals were digging every 30 s. Like us, they found that digging began as soon as the substrate was discovered, and that the animals worked quickly. No difference between males and females was observed, no more than two mole rats could stand side-by-side and dig simultaneously and conflict occurred during digging rotation. Task rotation was quick, dirt was swept to the toilet and not to the active nest box. However, we did observe them to sweep the debris to the food chamber, probably because of the spatial orientation. They found that larger individuals were the primary participants in volcanoing, which may reflect the positive effect of weight on exploration we observed. Moreover, because Lacey and Sherman did not have access to the accurate age of most colony members, they only used a relationship between mass, and age observed on captive individuals, or growth rate. We, on the other hand, had direct access to age for all colony members. The inverse effect of age and mass reveals two morphs participating in exploration/digging: young individuals and massive individuals. This supports the age polyethism hypothesis where animals specialize with age^5^, with young individuals involved in colony maintenance, and older (massive one) in colony defence. Indeed, we found that with all other parameters equal, younger individuals explored more, and for the same age, the heavier ones were the first to explore. This could be further explored in the future, as well as the effect of such behavioural modification on the disperser morph. Our experimental design could help explore how exploratory behaviour relates to the degree to which an animal expresses the disperser morph^7^ since all animals will express this morph at some point, but in captivity lose it and return to worker status.

The task of exploring could also be fulfilled by the young individuals, as part of their development, or due to their temperament^31^. Finally, data from laboratory settings may differ from field study as well, primarily because we cannot observe animals digging while they are underground. Our setting might thus be beneficial for future studies.

In conclusion, we found that the introduction of a diggable substrate increased the behavioural diversity and activity of one captive naked mole rat colony, as well as providing individuals with new decisions (digging or not). The use of such substrates could improve the welfare of naked mole rats under human care.

## Methods

### Ethical note

This study was approved by the Comité Cuvier of the MNHN (Muséum national d’Histoire naturelle, reference 2023-68-122) as a justified scientific project following the 3R principles. The authors confirm that all experiments were performed in accordance with relevant guidelines and regulations. This study was conducted in coordination with the animal care staff in order to respect the colony's behaviour and welfare, and to adapt our procedures, if needed, during the duration of the experiments. We were careful to minimise loud noises and vibrations that could cause disturbance to the colony. The captive colony was assembled in a box during the annual inventory, under the supervision of several veterinarians (Authorisation number: DTPP 2009-1002, Certificate of qualification no.: DTPP 2021-018 dated 8 January 2021) and the animal care staff. The individuals were captured by hand and handled individually for a health check (weighing, individual observations and injection of an RFID chip). The authors complied with the ARRIVE guidelines [].

### Subjects and housing

**Figure 5:**
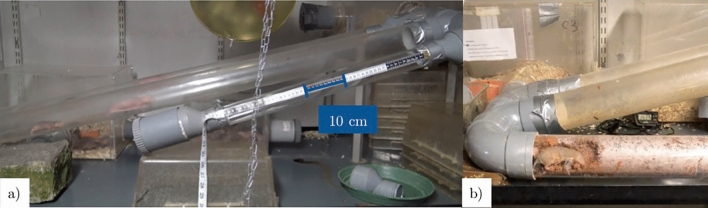
Housing of the naked mole rat colony. The naked mole rat colony was housed in a system of four boxes connected to one another directly, or through two central parallel tunnels, Tunnel 1 which was narrow, and Tunnel 2 which was wider.

We conducted our study on a captive colony of naked mole rats coming from the École nationale vétérinaire d’Alfort (EnvA), originating from Cape Nature, South Africa and which arrived in September 2021 at La Ménagerie, Zoo du Jardin des Plantes. An inventory of the colony was carried out on February 30th, 2023. The colony is composed of 79 individuals among which 40 males, 35 females, and 4 individuals whose sex could not be identified. All were more than a year old. During the inventory procedure, the veterinarians and curators weighed and identified individuals, and inserted radio-frequency identification (RFID) transponders in individuals that did not already possess one. The colony lives in a network of chambers interconnected by tunnels in Plexiglas®, with wood shaving bedding and kitchen paper. The light was on during the experiments since the colony is used to light on a daily basis during maintenance. The temperature is maintained at 28 °C with a 35% humidity so that temperature in the chambers is maintained between 28 and 30 °C, and 80% humidity (Fig. 5). Animals have access to food ad libitum with a base of tubers such as sweet potatoes at a rate of 6 g per individual and a mixture of vegetables at a rate of 3 g per individual (carrots, black radish, parsnip, pink radish, celeriac, red beetroot, black salsify, rutabaga, potato peas (Jicama), corn on the cob, green vegetables, carrot tops, butternut squash with seeds, aubergine; and in limited quantities: lettuce, cabbage, soya and beans).The environment is enriched with cardboard packaging to gnaw on. Each afternoon, keepers remove dirty bedding and occasionally wash the whole network with water. A radio is constantly playing in order to accustom animals to the sound of speech.

### Phase A: before substrate exposition

#### Exploratory behaviour

**Figure 6:**
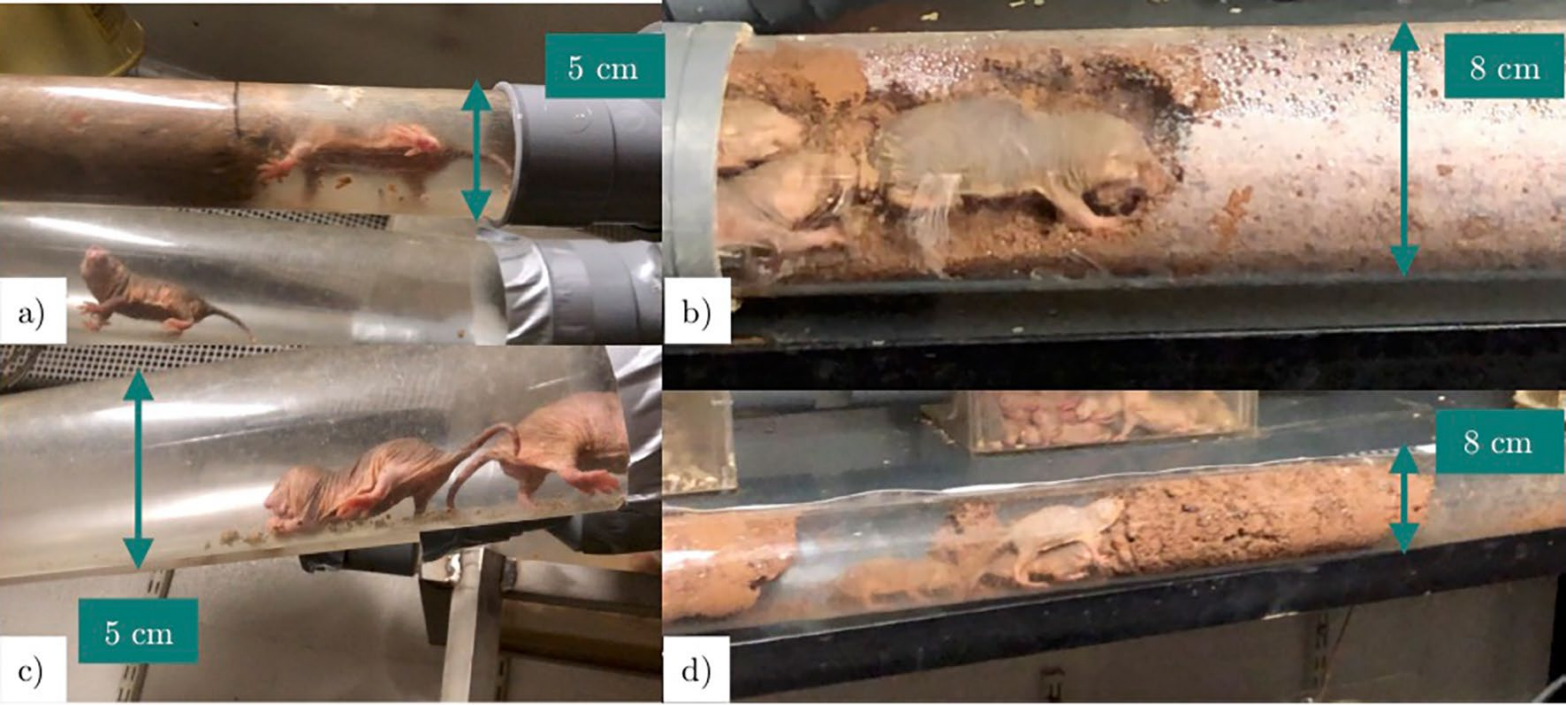
Tunnel installation for (**a**) exploration sessions, (**b**) observational sessions. The exploration tunnel was 58.5 cm long and the tunnel used during observational sessions was 140 cm long.

We chose not to test individuals separately from others in order to avoid the stress associated with isolation^32^. Rather, we let them choose whether or not they would participate and guaranteed them access to their habitat and other colony members. The exploration task consisted in introducing a 54 cm long and 5 cm diameter tunnel, parallel to their usual circuits with 17° inclination from the horizontal (Fig. 6). In order to limit the number of individuals using the tunnel, we chose a 5 cm diameter (to which they were already used to), and blocked one of the issues so that naked mole rats could only enter from one side: down-up (condition 1) or up-down (condition 2.a). We alternated between both conditions during one week, 1 h a day, 1 h per condition and per day. At the end of each test session, the tunnel was washed with water and black soap to remove the colony’s scent.

After the installation of the tunnel, each individual entering the tunnel was identified with a microchip reader. We considered an individual to have entered the tunnel when their four legs were inside the tunnel. The experiment was recorded with a Sony HD cam, and the entry times were taken into account by 2 different experimenters.

#### Gnawing frequency and entry flux of individuals

We performed 2 h long observations during which we counted the number of individuals in tunnel 1 and tunnel 2 each minute, and how many of them were gnawing on the tunnels.

### New substrates

For the first time, we introduced three different diggable substrates in our captive. We did not use a control colony, but rather compared the colony behaviour before and after exposition to these substrates. Like in the exploration tests, we used an up-down parallel tunnel filled with each kind of substrate. During these first two weeks of introduction, we observed one hour of interaction per substrates per week, for a total of 2 h per substrate. The order of exposition was randomly chosen and repeated two times. We used the reptile substrates described in Table S1. These first two weeks allowed us to make sure there was no issue with the substrate. Then, we continued the exposition to the substrates by adding tunnels of various lengths and diameters to allow the animals to dig for at least one hour a day. We adapted the duration of exposition and the type of substrate according to the time needed for the substrate to dry (see Table S2 for the planning). We made sure there were no problems introducing the substrate by talking to the animal care staff. On one occasion, they had to add water spray to increase the ambient humidity in the chambers. We also had to interrupt our sessions for a fortnight due to the birth of a litter by the queen. Unfortunately, a first session of "no substrate, upward angle" (the first test session) could not be analysed because data collection failure. We do not believe this session to be problematic since it does not drastically change our sample size and the behaviours observed during the 4 other sessions were quite homogenous.

### Phase B: after substrate exposition

We repeated the exploratory tests with an empty tunnel upwards (condition “post substrate, upward angle”), and a half-empty tunnel upwards (condition “substrate, upward angle”) to test the attractiveness of the substrate. We alternated with observation sessions as before, adding a third tunnel filled with substrate to test the effect of the presence of substrate on the colony behaviour.

### Data analysis

During one trial of condition “no substrate, upward angle”, we could not have access to the microchip identity number of explorers. We thus excluded this trial from analyses where individual identity was needed (*i.e. *Presence in the tunnels and Exploratory Latencies). All videos were analysed with knowledge of the condition tested.

#### Presence in the tunnels

We ran a GLMM model with a logit link function and binomial distribution (function “glmer”, package ‘lme4’ version 1.1-32^33^ to measure the effect of individual characteristics on presence during the different test phases. Fixed effects were mass, sex, age and condition, with an individual random effect. We checked that the model was correctly applied using the function check_model() designed for visual check of model assumption from the package ‘performance’ version 0.10.4^34^. As the sex of two individuals was unknown, they were excluded from this analysis for the generation of the null model only. We tested our model against the null model with a likelihood ratio test.

#### Number of explorers

We conducted a GLM analysis (function “glm”, package ‘stats’ version 4.2.3^35^), with the tunnel condition as a fixed effect, and using a Poisson family.

We checked that the model was correctly applied using the function check_model() from the package ‘performance’ version 0.10.4^34^. We tested our model against the null model with a likelihood ratio test.

#### Entry flux

We conducted a GLMM analysis (function “glmer”, package ‘lme4’ version 1.1-32^33^), with the tunnel condition as a fixed effect, including day, sessions, time interval and observation number (to correct for over-dispersion) as random effects, and using a Poisson family.

We checked that the model was correctly applied using the function check_model() from the package ‘performance’ version 0.10.4^34^. We tested our model against the null model with a likelihood ratio test.

#### Exploratory latencies

We adjusted a GLMM model with an identity link function and a gaussian distribution (function “lmer”, package ‘lme4’ version 1.1-32^33^) to measure the effect of individual characteristics on exploratory log transformed latencies during the different test phases. Fixed effects were mass, sex, age and test, with an individual random effect. “Sex” was then excluded because the effect was not significant and the model had a lower AIC. We checked that the model was correctly applied using the function check_model() from the package ‘performance’ version 0.10.4^34^. We tested our model against the null model with a likelihood ratio test.

#### Gnawing frequencies

We fitted a GLMM (function “glmer”, package ‘lme4’ version 1.1-32^33^) to model the number of individuals gnawing on the tunnel walls. Fixed effects were substrate exposition and tunnel condition. We included substrate condition (before or after substrate introduction) and tunnel condition as fixed effects, day and observation number (to correct for over-dispersion) as random effects, and used a binomial family. We checked that the model was correctly applied using the function check_model() from the package ‘performance’ version 0.10.4^34^.

All data analysis was conducted with R version 4.2.3.

### Supplementary Information

Supplementary Information 1.

Supplementary Information 2.

Supplementary Information 3.

Supplementary Information 4.

Supplementary Information 5.

Supplementary Information 6.

## Data Availability

Tables containing exploratory latencies and observation sessions are available as supplementary material.
