# Supplementary material for: Positive effect of a diggable substrate on the behaviour of a captive naked mole rat colony
Source: Sci Rep. 2024 Aug 29;14:20138. doi: 10.1038/s41598-024-64146-w (PMC11362154; doi:10.1038/s41598-024-64146-w)
Supplement: Supplementary file 5 — Supplementary Information 5. [file 41598_2024_64146_MOESM5_ESM.pdf]

Supplementary information.

Title

Positive effect of a diggable substrate on the behaviour of captive naked mole rats.

Authors

Myriam AMARI * [a,b] (ORCID: 0009-0001-3198-7341), Alma MARY[a] (ORCID: 0009-0009-1386-2156), Pauline ZABLOCKI-THOMAS[a,c] (ORCID: 0000-0002-2372-4760), Aude BOURGEOIS[d] (ORCID 0000-0003-3805-8374), Emmanuelle POUYDEBAT[a] (ORCID: 0000-0002-0542-975X)

Corresponding author

Myriam AMARI
• E-mail address: myriam.amari@ens.psl.eu

Affiliations

1.UMR 7179 CNRS/MNHN, Mécanismes Adaptatifs et Evolution, Muséum National d'Histoire Naturelle, 57 Rue Cuvier, 75231 Paris, France.
2.Département de Biologie, École normale supérieure, PSL Université Paris, F--75005, Paris, France.
3.Westfälische Wilhelms-Universität Münster, Germany
4.Ménagerie, Le Zoo du Jardin des Plantes, Muséum National d'Histoire Naturelle, 57 rue Cuvier, 75005 Paris, France

Correspondence

• Full postal address:
UMR 7179 MECADEV, CNRS/MNHN, Département Adaptations du Vivant, 57 Rue Cuvier, 75231 Paris, France
• Telephone +33 1 40 79 81 19

The supplementary materials comprise:
- The description of substrate characteristics
- Time schedule of the experiments
- Data of the colony
- Exploratory latencies before and after exposition to the substrate, as well as a table containing unique occurrences for all sessions (one entry per individual and per day).
- The observations of individuals gnawing on the walls¶

Table S1: Substrate characteristics according to the sellers' websites.

Substrate type	Description from the seller
Desert bedding, Lucky Retpile ® + Herp Pottery, Lucky Retpile ®	"Desert Bedding is a soil mixture developed in particular for desert and savannah type terrariums. In nature only few animals are living on sand, most are actually avoiding sand. Therefore it is better to use more natural substrates. Desert Bedding is recreating the soil in arid regions and has a high clay content which allows the animals to dig burrows. In the lower regions of the substrate the humidity can be kept higher which is important since a lot of desert reptiles like uromastyx can take up humidity over their skin."[1]
Terrano Acatama Ground, Hobby® + Herp Pottery, Lucky Retpile ®	"Mixing sand and humus with clay increases the stability of the resulting substrate and allows the animals to create burrows like they do in nature. Lucky Reptile Herp Pottery is a clay granulate. Its advantage over clay powder is that there is less dust during the mixing process. It can be mixed dry or dissolved in water. In the latter case the result is more homogenous. The more Herp Pottery you mix with the substrate the harder the result will be. Perfect for active and attractive terrarium sculpturing."[2]<br><br>"Terrano Atacama Ground is the ideal substrate for a natural terrarium design for desert and sand inhabitants. The particularly natural quality of the substrate and the clay content stimulates the animals' digging behaviour. With the Terrano Atacama Ground interesting landscapes, caves and rock formations can be recreated. The animals like to use the handmade superstructures as sunbathing areas and the dug-out caves as hiding places to cool off or sleep in. Terrano Atacama Ground is a natural substrate with no chemical colour additives."
Excavator ZooMed ®	"Excavator® Clay Substrate (XR-05) allows you to build a beautiful Naturalistic desert terrarium. Excavator® enables you to build burrows and tunnels similar to what is found in nature. These elements of a desert terrarium are very important and Excavator® makes it fun and easy to recreate! Excavator® can be used in a small corner of the terrarium, or throughout the entire terrarium and even as a background. This substrate encourages natural digging behaviours and can act as a form of enrichment for many desert species."[3]

1. Desert Bedding. *Lucky-Reptile* https://lucky-reptile.de/en/products/cage-substrate/substrate/325/desert-bedding.
2. Herp Pottery. *Lucky-Reptile* https://lucky-reptile.de/en/products/cage-substrate/sand-clay-and-gravel/338/herp-pottery.
3. HOBBY Terraristik. https://hobby-terraristik.com/EN/33204/terrano-atacama-ground.

Table S2: Test schedule. Durations noted with "~" are approximative because animals were left to interact with the enrichment until they finished it.

**Date**	**Test***	**Duration**
April 3, 2023	Condition "no substrate, upward angle" : no data could be collected	1h
April 4, 2023	Condition "no substrate, downward angle"	1h
April 5, 2023	Condition "no substrate, upward angle"	1h
April 6, 2023	Condition "no substrate, downward angle"	1h
April 7, 2023	Condition "no substrate, upward angle"	1h
April 11, 2023	Observation	2h
April 12, 2023	Observation	2h
April 13, 2023	Observation	2h
April 14, 2023	Observation	2h
April 17, 2023	Condition "no substrate, downward angle"	1h
April 18, 2023	Condition "no substrate, upward angle"	1h
April 19, 2023	Condition "no substrate, downward angle"	1h
April 20, 2023	Condition "no substrate, upward angle"	1h
Mai 9, 2023	Substrate explosion: Excavator ZooMed ®	1h30
	Substrate explosion: Terrano Atacama ®	~3h
Mai 10, 2023	Substrate explosion: Desert Bedding ®	~4h
Mai 11, 2023	Substrate explosion: Excavator ZooMed ®	~1h
Mai 12, 2023	Substrate explosion: Terrano Atacama ®	~3h
Mai 13, 2023	Substrate explosion: Desert Bedding ®	~3h
Mai 30, 2023	Substrate explosion: Desert Bedding ®	~3h
Mai 31, 2023	Substrate explosion: Terrano Atacama ®	~2h
June 1, 2023	Substrate explosion: Desert Bedding ®	~3h
June 2, 2023	Substrate explosion: Terrano Atacama ®	~3h
June 3, 2023	Substrate explosion: Terrano Atacama ®	~3h
June 5, 2023	Observation with Excavator ZooMed ®	2h
	Excavator ZooMed ®	1h
June 6, 2023	Condition "post substrate, upward angle"	1h
June 7, 2023	Observation with Excavator ZooMed ®	2h
June 8, 2023	Condition "substrate, upward angle"	1h
June 12, 2023	Observation with Desert Bedding ® and Terrano Atacama ®	2h
June 13, 2023	Condition "post substrate, upward angle"	1h
June 14, 2023	Observation with Desert Bedding ® and Terrano Atacama ®	2h
June 15, 2023	Condition "substrate, upward angle"	1h

*"Observation" refers to sessions where we observed and quantified the individuals' behaviours in the tunnels; "Observation with ..." refers to sessions where we observed and quantified the individuals' behaviours in the two main tunnels in presence of the mentioned substrate that were added in a third, wide horizontal tunnel.
